# Risk of somatic diseases in patients with eating disorders: the role of comorbid substance use disorders

**DOI:** 10.1017/S204579602200052X

**Published:** 2022-10-17

**Authors:** A. I. Mellentin, D. G. Nielsen, L. Skøt, R. K. Støving, M. M. Guala, A. S. Nielsen, R. Wesselhoeft, A. Mejldal

**Affiliations:** 1 Unit for Clinical Alcohol Research, Department of Clinical Research, University of Southern Denmark, Odense, Denmark; 2 Research Unit for Telepsychiatry and E-Mental Health, Center for Telepsychiatry, Region of Southern Denmark, Odense C, Denmark; 3 Brain Research-Inter-Disciplinary Guided Excellence (BRIDGE), Department of Clinical Research, University of Southern Denmark, Odense C, Denmark; 4 Center for Eating Disorders, Odense University Hospital, Odense C, Denmark; 5 Research Unit for Medical Endocrinology, Institute of Clinical Research, University of Southern Denmark, Odense C, Denmark; 6 Clinical Pharmacology, Pharmacy and Environmental Medicine, Department of Public Health, University of Southern Denmark, Odense C, Denmark; 7 Child and Adolescent Mental Health Odense, Mental Health Services in the Region of Southern Denmark, Odense C, Denmark; 8 Open Patient data Explorative Network, Odense University Hospital, Odense, Denmark

**Keywords:** Alcohol abuse, common mental disorders, health outcomes, prospective study

## Abstract

**Aims:**

Eating disorders (EDs) and substance use disorders (SUDs) often co-occur, and both involve somatic diseases. So far, no study has considered whether comorbid SUDs may impact somatic disease risk in patients with EDs. Therefore, this study aimed to examine the impact of comorbid SUDs on the risk of 11 somatic disease categories in patients with anorexia nervosa (AN), bulimia nervosa (BN) and unspecified eating disorder (USED) compared to matched controls.

**Methods:**

A retrospective cohort study was conducted using Danish nationwide registries. The study population included 20 759 patients with EDs and 83 036 controls matched on month and year of birth, sex and ethnicity. Hazard ratios (HRs) were calculated to compare the risk of being diagnosed with a somatic disease (within 11 categories defined by the ICD-10) following first ED diagnosis (index date) between ED patients and controls both with and without SUDs (alcohol, cannabis or hard drugs).

**Results:**

The ED cohort and matched controls were followed for 227 538 and 939 628 person-years, respectively. For ED patients with SUDs, the risk pattern for being diagnosed with different somatic diseases (relative to controls without SUDs) varied according to type of ED and SUD [adjusted HRs ranged from 0.95 (99% CI = 0.57; 1.59) to 4.17 (2.68, 6.47)]. The risk estimates observed among ED patients with SUDs were generally higher than those observed among ED patients without SUDs [adjusted HRs ranged from 1.08 (99% CI = 0.95, 1.22) to 2.56 (2.31, 2.84)]. Abuse of alcohol only had a non-synergistic effect on six disease categories in AN patients and five in BN and USED patients. Abuse of cannabis (with/without alcohol) had a non-synergistic effect on five disease categories in AN and BN patients and two in USED patients. Abuse of hard drugs (with/without alcohol or cannabis) had a non-synergistic effect on nine disease categories in AN patients, eight in BN patients and seven in USED patients.

**Conclusions:**

The present study documents non-synergistic but not synergistic harmful somatic consequences of SUDs among patients with different EDs, with AN and hard drugs being the most predominant factors. Hence, EDs and SUDs did not interact and result in greater somatic disease risk than that caused by the independent effects. Since EDs and SUDs have independent effects on many somatic diseases, it is important to monitor and treat ED patients for SUD comorbidity to prevent exacerbated physical damage in this vulnerable population.

## Introduction

Patients with eating disorders (EDs) may suffer from a range of somatic diseases, i.e. illnesses relating to the body (Momen *et al*., 2020, 2022), and mortality rates for EDs are approximately three times higher compared with the general population (Plana-Ripoll *et al*., 2019*a*; Mellentin *et al*., 2021*a*). Anorexia nervosa (AN) affects all somatic systems due to starvation, and purging behaviour adds to the risk of many conditions (including cardiovascular, dermatological, endocrine, gastrointestinal, haematological, neurological, musculoskeletal and genitourinary diseases) (Treasure *et al*., 2020). In bulimia nervosa (BN) and unspecified eating disorder (USED) (World Health Organization, 1992) or eating disorder not otherwise specified (EDNOS) (American Psychiatric Association, 2000), the physical damage due to starvation or vomiting is similar to that observed in AN, but usually less severe (Gibson *et al*., 2019; Treasure *et al*., 2020). Although studies indicate that AN has the most severe somatic consequences, a recent Danish population-based cohort study (Momen *et al*., 2022) found that hazard ratios (HRs) for a range of somatic diseases were quite similar in magnitude between patients with AN and patients with other EDs. However, the study did not examine BN and EDNOS separately, and there is a need for research examining the risk for somatic diseases across the full spectrum of EDs.

EDs frequently co-occur with substance use disorders (SUDs) (Bahji *et al*., 2019; Plana-Ripoll *et al*., 2019*b*; Mellentin *et al*., 2021*b*; Skøt *et al*., 2022). Alcohol use disorder (AUD) increases the risk for a large variety of somatic diseases (Rehm, 2011; Van Amsterdam *et al*., 2013), and the majority of somatic symptoms are also affected by chronic use of cannabis, stimulants and opiates (Devlin and Henry, 2008; Gordon, 2010; Van Amsterdam *et al*., 2013; Gordon *et al*., 2015). Furthermore, as may be the case for ED types, the risk pattern for being diagnosed with somatic diseases is likely to differ according to type of SUD (Van Amsterdam *et al*., 2013). Considering that independent of each other, EDs and SUDs are associated with various somatic diseases, it could be speculated that comorbid SUDs may have non-synergistic or even positive synergistic harmful effects on somatic morbidity in ED patients. A non-synergistic effect would occur when EDs and SUDs independently contribute to overall somatic morbidity, whereas a positive synergistic effect would occur when EDs and SUDs interact resulting in the combination effect being greater than the independent effects. So far, this clinically relevant issue has not been addressed. The aim of this study was thus to examine the impact of comorbid SUDs (alcohol, cannabis and hard drugs) on the risk of 11 somatic disease categories in patients with AN, BN and USED compared to matched controls.

## Methods

The reporting of this study is in accordance with the Strengthening the Reporting of Observational Studies in Epidemiology (STROBE) guidelines (von Elm *et al*., 2008).

### Study design and population

This study is based on a nationwide cohort of all individuals born in Denmark after 31 December 1967, who were diagnosed with an ED according to the International Classification of Diseases, 10th version (ICD-10) (World Health Organization, 1992) at an in- or outpatient treatment facility for EDs between 1 January 1994 and 31 December 2015. Data were obtained from nationwide registers. The registers were linked using the unique personal identification number assigned to all permanent residents in Denmark since 1968, which is part of the personal information stored in the Danish Civil Registration System (DCRS) (Pedersen, 2011).

The Danish National Patient Register (DNPR) (Lynge *et al*., 2011) was used to create the cohort of ED patients and it was further divided into three subcohorts: (1) AN (ICD-10 codes F50.0, AN; F50.1, atypical AN); (2) BN (codes F50.2, BN; F50.3, atypical BN); and (3) USED (codes F50.8, other EDs; F50.9, eating disorder, unspecified). The F50.8 category comprises patients with binge-eating disorder or other disorders such as selective eating disorder, avoidant-restrictive food intake disorder or pica. The F50.9 category comprises patients who do not fulfil the criteria for AN or BN, such as those presenting with subthreshold symptoms of AN or BN, mixed features of AN or BN or extremely atypical ED (not characterised by either disorder). The index date was the date of first ED diagnosis. Exclusion criteria were (1) registration of an ICD-8 ED diagnosis (codes 306.50, 306.58, 306.59) between 1976 and 1994 (to ensure that the cohort only comprised patients with a first ED diagnosis)^1^; (2) ED diagnosis registered before eight years of age (to reduce misclassification, e.g. feeding disorders in infancy and childhood); and (3) emigration between the index date and termination of the inclusion period. If a patient had received more than one ED diagnosis during the inclusion period, we used the first registered diagnosis. Moreover, if a patient had been registered with two ED diagnoses on the same day, we applied the following hierarchy: (1) AN and BN or USED was coded as AN; (2) BN and USED was coded as BN. Information on diagnostic crossover can be found in online Supplementary Table S1. Over 70% of patients in each ED group did not cross-over to another ED during the observation period.

The control group, identified via the DCRS (Pedersen, 2011), included individuals from the general population (selected in a 1:4 ratio) who had (1) no registered ICD-8 ED diagnosis between 1976 and 1994 or ICD-10 ED diagnosis between 1994 and 31 December 2015; and (2) not emigrated between the index date and the end of the inclusion period. Controls were matched to ED cases by month and year of birth, sex (male or female) and ethnicity (Danish or immigrant/descendant), and they were allocated the same index date as their matched case. Data on birth date, sex, ethnicity and cohabitation status (cohabiting, living alone or under age 18 and living with a caregiver) at the index date were extracted from the DCRS (Pedersen, 2011). We used the Danish Education Register (Jensen and Rasmussen, 2011) and Danish Income Statistics Register (Employment Classification Module) (Baadsgaard and Quitzau, 2011) to obtain data on highest achieved education (primary/unknown, lower secondary or upper secondary or higher) and employment status (employed, unemployed or other), respectively. Information on the original classifications of cohabitation status, highest achieved education, and employment status is provided in online Supplementary file S1.

Study participants were followed from the index date until the date of first somatic disorder diagnosis, death, emigration or end of the study period (31 December 2018), whichever came first. Dates of death were obtained from the Danish Register of Causes of Death (Helweg-Larsen, 2011) and dates of emigration from the DCRS (Pedersen, 2011).

### Substance use disorders

Study participants were classified as having a SUD diagnosis if they had been registered in the DNPR (Lynge *et al*., 2011) or Psychiatric Central Research Register (Mors *et al*., 2011) with a relevant ICD-10 code from one of the following eight categories: alcohol (F10.1 & F10.2); opioids (F11.1 & F11.2); cannabis (F12.1 & F12.2); sedatives/hypnotics (F13.1 & F13.2); cocaine (F14.1 & F14.2); other stimulants excluding caffeine (F15.1 & F15.2); hallucinogens (F16.1 & F16.2); volatile solvents (F18.1 & F18.2); multiple drug use and use of other substances (F19.1 & F19.2). Study participants registered as having undergone SUD (illegal drugs only) treatment in the Register of Substance Abusers in Treatment (1996–2018) (Sundhedsdatastyrelsen, 2022) or AUD treatment in the National Alcohol Treatment Register (2007–2018) (Schwarz *et al*., 2018), were also considered to have a SUD diagnosis. Study participants diagnosed with SUDs were included regardless of whether registration of the SUD diagnosis occurred before or after the index date. The reason this procedure was chosen is that development of SUDs is likely to have started before treatment initiation. SUDs were grouped into alcohol, cannabis or hard drugs (heroin and other opioids, sedatives/hypnotics, cocaine and other stimulants, multiple substances and other psychoactive substances, e.g. hallucinogens, volatile solvents, designer drugs etc.).

### Outcome: somatic diseases

Information on first somatic diagnosis after the index date was obtained from the DNPR (Lynge *et al*., 2011). Individual diagnostic codes were grouped into the following 11 broad categories in accordance with the chapters in the ICD-10 (World Health Organization, 1992): infectious (A00-B99); neoplasms (C00-D48); haematological (D50-D89); endocrine (E00-E90); neurological (G00-H95); circulatory (I00-I99); respiratory (J00-J99); gastrointestinal (K00-K93); dermatological (L00-L99); musculoskeletal (M00-M99); and genitourinary (N00-N99). If study participants had more than one somatic diagnosis, we used the first registered.

### Statistical analysis

First, we compared sociodemographic and clinical characteristics (1) between patients with AN, BN or USED and respective matched controls and (2) across the ED types, using chi-square tests, t tests or analysis of variance. Second, we used Cox regression (HRs) and 99% confidence intervals (CIs)] to calculate the risk of being diagnosed with a somatic disorder (within 11 categories) after the index date in stratified analysis by ED type. We compared patients with AN, BN or USED with and without SUDs to respective matched controls without SUDs, adjusting for age at the index date, sex and birth year. The same analyses were used to compare patients with AN, BN or USED with SUDs to patients with the corresponding ED but without SUDs. The following SUD categories were examined: (1) alcohol only; (2) cannabis (with/without alcohol); and (3) hard drugs (with/without alcohol or cannabis). Third, we tested the possibility of an interaction effect between case status (0 = control, 1 = case) and type of SUD using the likelihood-ratio test. Interactions were included if the likelihood-ratio test suggested a better fit (*p* < 0.005). Lastly, we estimated the cumulative incidence of being diagnosed with a somatic disorder (within 11 categories) after the index date in patients with AN, BN or USED, comparing the rates with respective controls. ED patients and controls were divided into groups with and without SUDs (results are shown in online Supplementary Fig. S1).

## Results

### Characteristics of the study population

The overall ED cohort (*n* = 20 759) and matched controls (*n* = 83 036) were followed for 227 538 and 939 628 person-years, respectively. Table 1 compares sociodemographic and clinical characteristics between patients with AN (*n* = 8108), BN (*n* = 5485) or USED (*n* = 7166) and respective matched controls.

**Table 1.** Comparison of sociodemographic and clinical characteristics between patients with AN, BN or USED and respective matched controls

	AN patients (*N* = 8108)		AN controls (*N* = 32 432)			BN patients (*N* = 5485)		BN controls (*N* = 21 940)			USED patients (*N* = 7166)		USED controls (*N* = 28 664)		
*Sociodemographic characteristics*															
	*N*	%	*N*	%	*p*	*N*	%	*N*	%	*p*	*N*	%	*N*	%	*p*
Age group^a^					1.00					1.00					1.00
<18 years	4117	50.8	16 468	50.8		836	15.2	3344	15.2		3107	43.4	12 428	43.4	
18–30 years	3605	44.5	14 420	44.5		4085	74.0	16 232	74.0		3295	46.0	13 180	46.0	
>30 years	386	4.8	1544	4.8		591	10.8	2364	10.8		764	10.7	3056	10.7	
Sex^a^					1.00					1.00					1.00
Male	514	6.3	2056	6.3		127	2.3	508	2.3		774	10.8	3096	10.8	
Female	7594	93.7	30 376	93.7		5358	97.7	21 432	97.7		6392	89.2	25 568	89.2	
Ethnicity^a^					1.00					1.00					1.00
Danish	7694	94.9	30 776	94.9		5188	94.6	20 752	94.6		6681	93.2	26 724	93.2	
Immigrant or descendent	414	5.1	1656	5.1		297	5.4	1188	5.4		485	6.8	1940	6.8	
Cohabiting status					<0.001					<0.001					<0.001
Cohabiting	1847	22.8	9574	29.5		1825	33.3	11 020	50.2		1784	24.9	9891	34.5	
Living alone	2139	26.4	6390	19.7		2820	51.5	7576	34.5		2270	31.7	6345	22.1	
Under age 18 and living with a caregiver	4117	50.8	16 468	50.8		836	15.3	3344	15.2		3107	43.4	12 428	43.4	
Highest achieved education					<0.001					0.48					<0.001
Primary/unknown	2351	29.2	9449	29.3		287	5.3	1106	5.1		1821	25.5	7162	25.2	
Lower secondary	3968	49.2	15 035	46.6		2420	44.4	9857	45.3		3482	48.7	12 313	43.2	
Upper secondary or higher	1745	21.6	7749	24.0		2744	50.3	10 806	49.6		1841	25.8	8998	31.6	
Employment status at the index date					<0.001					<0.001					<0.001
Employed	1179	14.5	6594	20.3		1.684	30.7	8917	40.6		1258	17.6	7685	26.8	
Unemployed	675	8.3	1304	4.0		701	12.8	1761	8.0		921	12.9	1326	4.6	
Other	6254	77.1	24 534	75.6		3100	56.5	11 262	51.3		4.987	69.6	19 653	68.6	
	Mean	s.d.	Mean	s.d.	*p*	Mean	s.d.	Mean	s.d.	*p*	Mean	s.d.	Mean	s.d.	*p*
Age (years)^a^	18.8	5.7	18.8	5.7	1.00	23.0	5.7	23.0	5.7	1.00	20.3	7.2	20.3	7.2	1.00
*Substance use disorder characteristics*															
	*N*	%	*N*	%	*p*	*N*	%	*N*	%	*p*	*N*	%	*N*	%	*p*
Any SUD	676	8.3	917	2.8	<0.001	602	11.0	568	2.6	<0.001	815	11.4	791	2.8	<0.001
Alcohol abuse/dependence	305	3.8	312	1.0	<0.001	332	6.1	249	1.1	<0.001	345	4.8	274	1.0	<0.001
Cannabis abuse/dependence	298	3.7	453	1.4	<0.001	194	3.5	231	1.1	<0.001	394	5.5	381	1.3	<0.001
Hard drug abuse/dependence^b^	299	3.7	426	1.3	<0.001	281	5.1	275	1.3	<0.001	387	5.4	366	1.3	<0.001
Timing of SUD diagnosis^c^					<0.001					<0.001					<0.001
SUD predates ED by more than 1 year	131	19.4	155	16.9		109	18.1	181	31.9		195	23.9	209	26.4	
SUD and ED diagnosed within the same year	144	21.3	95	10.4		154	25.6	51	9.0		226	27.7	95	12.0	
ED predates SUD by more than 1 year	401	59.3	667	72.7		339	56.3	336	59.2		394	48.3	487	61.6	
	Mean	s.d.	Mean	s.d.	*p*	Mean	s.d.	Mean	s.d.	*p*	Mean	s.d.	Mean	s.d.	*p*
Age at first SUD diagnosis (years)															
Alcohol abuse/dependence	26.6	8.1	26.1	7.7	0.48	29.1	7.6	29.3	8.0	0.74	26.6	7.7	25.9	8.1	0.31
Cannabis abuse/dependence	22.4	5.5	21.4	5.3	0.016	23.7	5.8	24.0	6.1	0.62	22.2	5.8	21.4	5.0	0.062
Hard drug abuse/dependence	23.0	6.3	22.5	5.7	0.30	25.2	7.0	24.5	6.5	0.27	23.6	6.3	22.5	5.2	0.006
*ICD-10 somatic disorders*															
	*N*	%	*N*	%	*p*	*N*	%	*N*	%	*p*	*N*	%	*N*	%	*p*
At least one somatic disorder after the index date	7408	91.4	27 491	84.8	<0.001	5180	94.4	19 827	90.4	<0.001	6564	91.6	23 466	81.9	<0.001
Infectious diseases (A00-B99)	950	11.7	2437	7.5	<0.001	710	12.9	1632	7.4	<0.001	810	11.3	1908	6.7	<0.001
Neoplasms (C00-D48)	675	8.3	2221	6.8	<0.001	594	10.8	2193	10.0	0.067	518	7.2	1823	6.4	0.008
Haematological diseases (D50-D89)	252	3.1	560	1.7	<0.001	164	3.0	475	2.2	<0.001	217	3.0	433	1.5	<0.001
Endocrine diseases (E00-E90)	1038	12.8	2177	6.7	<0.001	759	13.8	1854	8.5	<0.001	1060	14.8	1762	6.1	<0.001
Neurological diseases (G00-H95)	1401	17.3	4256	13.1	<0.001	1128	20.6	3304	15.1	<0.001	1395	19.5	3397	11.9	<0.001
Circulatory diseases (I00-I99)	629	7.8	1603	4.9	<0.001	539	9.8	1575	7.2	<0.001	516	7.2	1332	4.6	<0.001
Respiratory diseases (J00-J99)	1229	15.2	3995	12.3	<0.001	866	15.8	2650	12.1	<0.001	1135	15.8	3000	10.5	<0.001
Gastrointestinal diseases (K00-K93)	1940	23.9	5396	16.6	<0.001	1454	26.5	4122	18.8	<0.001	1795	25.0	4255	14.8	<0.001
Dermatological diseases (L00-L99)	1044	12.9	2787	8.6	<0.001	739	13.5	2128	9.7	<0.001	837	11.7	2215	7.7	<0.001
Musculoskeletal diseases (M00-M99)	2402	29.6	7624	23.5	<0.001	1732	31.6	5706	26.0	<0.001	2038	28.4	5924	20.7	<0.001
Genitourinary diseases (N00-N99)	2440	30.1	7599	23.4	<0.001	2063	37.6	6793	31.0	<0.001	2087	29.1	6114	21.3	<0.001
*Follow-up*															
	Median	IQR	Median	IQR	*p*	Median	IQR	Median	IQR	*p*	Median	IQR	Median	IQR	*p*
Follow-up time (years)	10.5	6.5, 16.1	10.6	6.5, 16.2	0.29	12.2	7.1, 18.0	12.2	7.2, 18.1	0.51	8.6	5.4, 13.7	8.7	5.5, 13.9	0.11
*Mortality*															
	*N*	%	*N*	%	*p*	*N*	%	*N*	%	*p*	*N*	%	*N*	%	*p*
Deaths during follow-up	115	1.4	114	0.4	<0.001	53	1.0	117	0.5	<0.001	126	1.8	92	0.3	<0.001
	Mean	s.d.	Mean	s.d.	*p*	Mean	s.d.	Mean	s.d.	*p*	Mean	s.d.	Mean	s.d.	*p*
Age at death	30.1	8.5	29.9	8.3	0.83	30.7	7.7	32.7	7.5	0.12	30.9	8.7	30.2	9.7	0.57

AN, anorexia nervosa; BN, bulimia nervosa; IQR, interquartile range; s.d., standard deviation; SUD, substance use disorder; USED, unspecified eating disorder.

a Matching variable.

b The hard drugs category includes heroin and other opioids, sedative-hypnotics, cocaine and other stimulants, multiple substances and other psychoactive substances (e.g. hallucinogens, volatile solvents and designer drugs).

c The timing of first SUD diagnosis among individuals in the control group was determined from the index date of the eating disorder patients to whom they were matched.

At study entry, the highest age was observed in BN patients (mean = 23.0 years), followed by USED patients (mean = 20.3 years), then AN patients (mean = 18.8 years). The majority of patients in each ED group were female (89.2–97.7%) and Danish (92.3–94.9%). Compared to controls, a significantly higher proportion of patients in each ED group were diagnosed with alcohol, cannabis and hard drug abuse/dependence. Furthermore, significantly more patients in each ED group were diagnosed with a somatic disorder in each of the 11 categories, the only exception being neoplasms in the BN group (*p* = 0.067). The most common somatic diseases in all ED groups were genitourinary, musculoskeletal and gastrointestinal diseases (see Table 1).

A comparison of characteristics across the ED types is presented in online Supplementary Table S2.

### Risk of somatic diseases

**Fig. 1.**
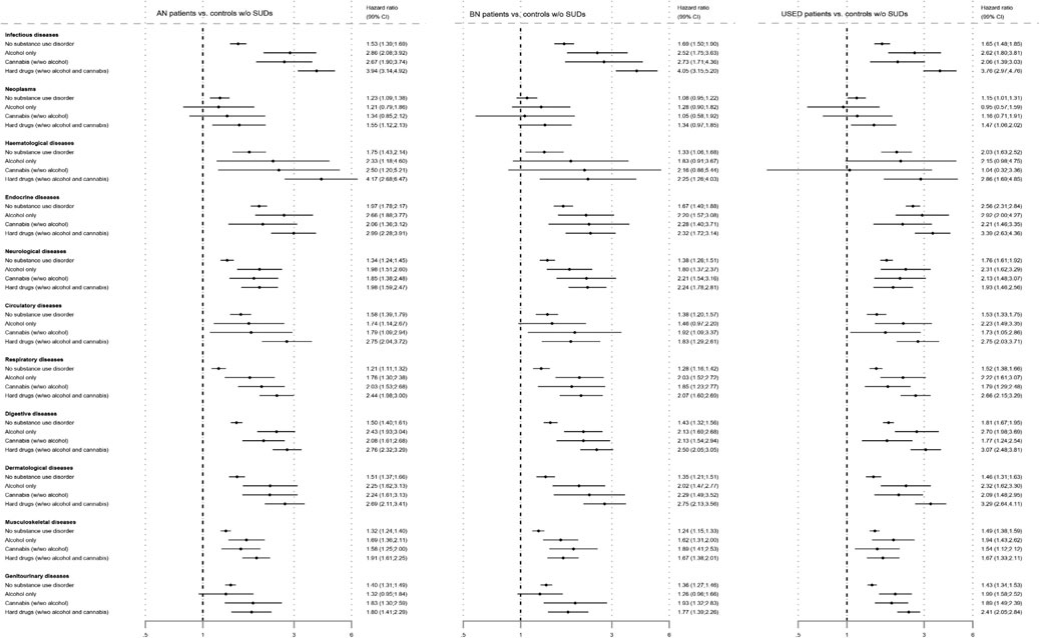
Adjusted HRs for the risk of being diagnosed with a somatic disease (within 11 categories) in patients with anorexia nervosa (AN), bulimia nervosa (BN) or unspecified eating disorder (USED) with and without substance use disorders (SUDs) compared to matched controls without SUDs.

Figure 1 shows the adjusted HRs (aHRs) for the risk of being diagnosed with a somatic disease (within 11 categories) in patients with AN, BN or USED with and without SUDs compared to controls without SUDs.

In the AN group, an elevated risk (compared to controls without SUDs) was observed for (1) all disease categories among AN patients without SUDs (aHRs ranging from 1.21 for respiratory diseases to 1.97 for endocrine diseases); (2) nine disease categories among AN patients who abused alcohol alone (aHRs ranging from 1.69 for musculoskeletal diseases to 2.86 for infectious diseases; no increased risk observed for neoplasms and genitourinary diseases); (3) ten disease categories among AN patients who abused cannabis (with/without alcohol) (aHRs ranging from 1.58 for musculoskeletal diseases to 2.67 for infectious diseases; no increased risk observed for neoplasms); and (4) all disease categories among AN patients who abused hard drugs (with/without alcohol or cannabis) (aHRs ranging from 1.55 for neoplasms to 4.17 for haematological diseases).

In the BN group, an elevated risk (compared to controls without SUDs) was observed for (1) ten disease categories among BN patients without SUDs (aHRs ranging from 1.24 for musculoskeletal diseases to 1.69 for infectious diseases; no increased risk observed for neoplasms); (2) seven disease categories among BN patients who abused alcohol alone (aHRs ranging from 1.62 for musculoskeletal diseases to 2.52 for infectious diseases; no increased risk observed for neoplasms and haematological, circulatory and genitourinary diseases); (3) nine disease categories among BN patients who abused cannabis (with/without alcohol) (aHRs ranging from 1.85 for respiratory diseases to 2.73 for infectious diseases; no increased risk observed for neoplasms and haematological diseases); and (4) ten disease categories among BN patients who abused hard drugs (with/without alcohol or cannabis) (aHRs ranging from 1.67 for musculoskeletal diseases to 4.05 for infectious diseases; no increased risk observed for neoplasms).

In the USED group, an elevated risk (compared to controls without SUDs) was observed for (1) all disease categories among USED patients without SUDs (aHRs ranging from 1.15 for neoplasms to 2.56 for endocrine diseases); (2) nine disease categories among USED patients who abused alcohol alone (aHRs ranging from 1.94 for musculoskeletal diseases to 2.92 for endocrine diseases; no increased risk observed for neoplasms and haematological diseases); (3) nine disease categories among USED patients who abused cannabis (with/without alcohol) (aHRs ranging from 1.54 for musculoskeletal diseases to 2.21 for endocrine diseases; no increased risk observed for neoplasms and haematological diseases); (4) all disease categories among USED patients who abused hard drugs (with/without alcohol or cannabis) (aHRs ranging from 1.47 for neoplasms to 3.76 for infectious diseases).

**Fig. 2.**
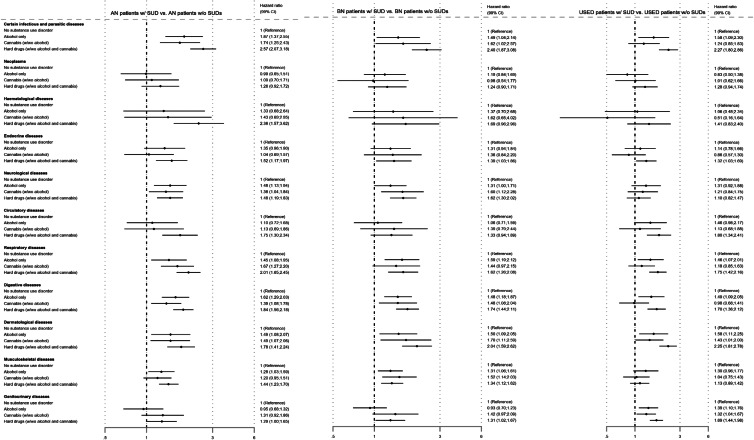
Adjusted HRs for the risk of being diagnosed with a somatic disease (within 11 categories) in patients with anorexia nervosa (AN), bulimia nervosa (BN) or unspecified eating disorder (USED) with substance use disorders (SUDs) compared to respective eating disorder patients without SUDs.

Figure 2 shows the HRs for the risk of being diagnosed with a somatic disease (within 11 categories) in patients with AN, BN or USED with SUDs compared to respective ED patients without SUDs.

SUDs had non-synergistic harmful effects on somatic morbidity in ED patients. The magnitude of the non-synergistic effects did not differ greatly across the groups.

Abuse of alcohol only had a non-synergistic effect on six disease categories in the AN group (infectious, neurological, respiratory, gastrointestinal, dermatological, musculoskeletal) and five in the BN (infectious, respiratory, gastrointestinal, dermatological, musculoskeletal) and USED (infectious, respiratory, gastrointestinal, dermatological, genitourinary) groups.

Abuse of cannabis (with/without alcohol) had a non-synergistic effect on five disease categories in the AN (infectious, neurological, respiratory, gastrointestinal, dermatological) and BN (infectious, neurological, gastrointestinal, dermatological, musculoskeletal) groups and two in the USED group (dermatological, genitourinary).

Abuse of hard drugs (with/without alcohol or cannabis) had a non-synergistic effect on nine disease categories in the AN group (infectious, haematological, endocrine, neurological, circulatory, respiratory, gastrointestinal, dermatological, musculoskeletal), eight in the BN group (infectious, endocrine, neurological, respiratory, gastrointestinal, dermatological, musculoskeletal, genitourinary) and seven in the USED group (infectious, endocrine, circulatory, respiratory, gastrointestinal, dermatological, genitourinary).

Furthermore, there was a negative synergistic effect of (1) abuse of alcohol alone on genitourinary diseases in the AN and BN groups, (2) abuse of cannabis (with/without alcohol) on gastrointestinal diseases in the USED group and (2) abuse of hard drugs (with/without alcohol or cannabis) on genitourinary diseases in the AN group as well as on neurological and musculoskeletal diseases in the USED group (see online Supplementary Table S3). In these cases, the combined effect is lower than the sum of the individual effects.

## Discussion

In this retrospective cohort study, we examined the contribution of comorbid SUDs to somatic disease risk in patients with different EDs (*n* = 20 759) compared to match controls (*n* = 83 036). Among ED patients with SUDs, the risk pattern for being diagnosed with different somatic diseases (compared to controls without SUDs) varied according to type of ED and SUD. The risk estimates observed among ED patients with SUDs were generally higher than those observed among ED patients without SUDs. Our finding that ED patients, including those with and without SUDs, have a higher risk for a variety of somatic diseases compared to controls without SUDs expand those of Momen and colleagues (Momen *et al*., 2022) by highlighting risk patterns for being diagnosed with different somatic diseases among patients across the full spectrum of EDs both with and without SUDs. Of more interest in the context of the current study is that SUDs were found to exert non-synergistic harmful effects on somatic morbidity in ED patients.

Abuse of alcohol alone had a non-synergistic effect on (1) infectious, respiratory, gastrointestinal and dermatological diseases in all ED groups; (2) musculoskeletal diseases in the AN and BN groups; (3) neurological diseases in the AN group; and (4) genitourinary diseases in the USED group. Furthermore, abuse of cannabis (with/without alcohol) had a non-synergistic effect on (1) dermatological diseases in all ED groups; (2) infectious, gastrointestinal and neurological diseases in the AN and BN groups; (3) respiratory diseases in the AN group; (4) musculoskeletal diseases in the BN group; and (5) genitourinary diseases in the USED group. Moreover, there was a non-synergistic effect of hard drugs (with/without alcohol or cannabis) on (1) infectious, respiratory, gastrointestinal, dermatological and endocrine diseases in all ED groups; (2) neurological and musculoskeletal diseases in the AN and BN groups; (3) circulatory diseases in the AN and USED groups; (4) genitourinary diseases in the BN and USED groups; and (5) haematological diseases in the AN group.

The observed non-synergistic effects are not unexpected given the existing evidence linking EDs and SUDs independently to many somatic diseases (Devlin and Henry, 2008; Gordon, 2010; Rehm, 2011; Gordon *et al*., 2015; Gibson *et al*., 2019; Treasure *et al*., 2020; Momen *et al*., 2022). Regarding infectious diseases, it is well known that patients with EDs or SUDs have poor nutritional state and an affected immune system (Ross *et al*., 2012; Raevuori *et al*., 2016; Vold *et al*., 2019). Abuse of alcohol, cannabis and hard drugs (e.g. cocaine and opioids) modulates the immune system leading to insufficient immune response (Imtiaz *et al*., 2017; Magel *et al*., 2020; Maggirwar and Khalsa, 2021). Several respiratory and dermatological diseases may be associated with an impaired function of the immune system (Richmond and Harris, 2014; Patrawala *et al*., 2020). Respiratory tract diseases are common in ED patients (Tenholder and Pike, 1991; Birmingham *et al*., 2003; Brown *et al*., 2005; Treasure *et al*., 2020; Grayeb *et al*., 2021). Alcohol abuse has been linked to pneumonia and acute respiratory distress syndrome (Kershaw and Guidot, 2008; Ross *et al*., 2012; Mehta and Guidot, 2017). Smoking cannabis can have a similar effect on the airways and cause similar respiratory diseases as smoking tobacco, e.g. chronic obstructive lung disease (Tashkin, 2018). Inhalation of drugs like cocaine and opioids can cause damage to the respiratory tract constituted by e.g. thermal burns (Nanayakkara and McNamara, 2020). Opioids alone (Wang *et al*., 2021), but also in combination with benzodiazepines (Durkin, 2016) affect the physiologic functions of the respiratory system (Wang *et al*., 2021). Some dermatological diseases such as xerosis and acne occur frequently in ED patients (Glorio *et al*., 2000; Strumia, 2005). Patients who abuse alcohol have an increased risk of inflammatory skin diseases (Al-Jefri *et al*., 2017), and abusers of hard drugs may experience skin infection (Kaushik *et al*., 2011). Also, cannabis abuse has been linked to increased risk of acne and candidiasis (Shao *et al*., 2021).

Regarding gastrointestinal diseases, ED patients exhibiting purging behaviour often experience oral cavity problems, and oral diseases are also common in patients with SUDs (Rossow, 2021). Alcohol abuse is associated with several acute and chronic diseases in the digestive system, e.g. bleedings (Kelly *et al*., 1995), pancreatitis (Lankisch *et al*., 2015) and liver diseases (Osna *et al*., 2017). Cannabis abuse may cause the relatively new disorder, cannabinoid hyperemesis syndrome, which can instigate several symptoms from the digestive system (Nasser *et al*., 2020). Abuse of cocaine can damage the intestines (Chivero *et al*., 2019) and cause haemorrhage in the digestive system (Carlin *et al*., 2014). Opioids in general affect the gastrointestinal system e.g. by reducing motility leading to constipation (Leppert, 2012).

Malnutrition is an endocrine disease, is common among patients with EDs or SUDs (Warren, 2011; Ross *et al*., 2012; Misra and Klibanski, 2014; Wiss *et al*., 2019; Skowron *et al*., 2020; Mahboub *et al*., 2021), and is also associated with the development of many other diseases (Usdan *et al*., 2008; Ross *et al*., 2012; Dobner and Kaser, 2018; Rajamanickam *et al*., 2020). Wernicke-Korsakoff's syndrome is caused by malnutrition due to e.g. excessive alcohol consumption (Praharaj *et al*., 2021) and this disease has also been observed in ED patients, especially those with AN (Oudman *et al*., 2018). Malnutrition can also cause type 2 diabetes and metabolic syndrome, which are common among substance users (Nabipour *et al*., 2014; Ojo *et al*., 2018). Lower levels of the thyroid hormones thyroxine and triiodothyronine are common in AN patients (Warren, 2011; Støving, 2019) and among opioid users (De Vries *et al*., 2020).

Several neurological diseases such as polyneuropathies and seizures are associated with malnutrition (Patchell *et al*., 1994) which, as previously mentioned, has been linked to both EDs and SUDs. In AN patients, it has been shown that 47% had neurological complications and 21% had more than one (Patchell *et al*., 1994). Neuropathies and epilepsy are common in patients with AN (Patchell *et al*., 1994) or BN (Rushing *et al*., 2003) and also among patients with SUDs (Ross *et al*., 2012; Nasser *et al*., 2020). Furthermore, discontinuing the use of alcohol (Rogawski, 2005), benzodiazepines (Soyka, 2017) and hard drugs such as cocaine (Koppel *et al*., 1996) and heroin (Saboory *et al*., 2007) may cause seizures.

Some circulatory and vascular diseases are common among ED patients. A recent study showed that some AN patients have a non-beneficial profile of lipids (Hussain *et al*., 2019). A similar condition is found among obese patients (Vekic *et al*., 2019), and obesity is common in some types of USED (Gormally *et al*., 1982; Mitchell *et al*., 2015; Di Giacomo *et al*., 2022). Dyslipidemia is associated with an increased risk of cardiovascular diseases (Vekic *et al*., 2019), which are often seen in cocaine users (Talarico *et al*., 2017). Other cardiovascular diseases that have been linked to SUDs are diseases in both superficial and deep veins due to intravenous administration of drugs (Jain *et al*., 2021) and infection (Pieper *et al*., 2007; Straw *et al*., 2020).

As for genitourinary diseases, young women with EDs tend to have a greater number of sexual partners and a lower use of condoms (Fergus *et al*., 2019), which may increase the risk of sexually transmitted diseases. Also, infertility, both male and female, is common among patients who are underweight or obese (Zain and Norman, 2008; Katib, 2015; Guo *et al*., 2019; Boutari *et al*., 2020). Furthermore, abuse of alcohol, cannabis and hard drugs can affect fertility in both males and females (Sansone *et al*., 2018; De Angelis *et al*., 2020).

Although non-synergistic effects were observed in all ED groups, the most disease categories were affected in the AN group, which is interesting given that patients in the AN group were younger than those in the BN and USED groups (and therefore had less time to develop SUDs and somatic diseases) and the burden of somatic diseases increases with age. It has also been shown in numerous studies that AN has the most severe somatic consequences (Treasure *et al*., 2020), although prior studies did not take comorbid SUDs into consideration. Thus, the combination of AN and SUDs may be particularly physically damaging. Regarding type of SUD, abuse of hard drugs (with/without alcohol or cannabis) had a non-synergistic effect on more somatic disease categories across ED types than abuse of alcohol only and cannabis (with/without alcohol). A prior study by Van Amsterdam and colleagues (Van Amsterdam *et al*., 2013) examined the impact of chronic use of different legal and illegal substances on somatic diseases and found that hard drugs are the most physically damaging substances, which may explain the observed pattern.

Contrary to what could be expected, we did not find any positive synergistic effects of SUDs on somatic diseases, i.e. that the interaction between EDs and SUDs increased and catalysed the influence of SUDs beyond the independent effects. Instead, we found that abuse of alcohol alone had a negative synergistic effect on genitourinary diseases in the AN and BN groups, and there was also a negative synergistic effect of cannabis (with/without alcohol) on gastrointestinal diseases in the USED group. Furthermore, abuse of hard drugs (with/without alcohol or cannabis) had a negative synergistic effect on genitourinary diseases in the AN group as well as on neurological and musculoskeletal diseases in the USED group. These findings indicate that the combined effect of certain EDs and SUDs is smaller than the sum of the individual effects. Although this could suggest that it less harmful to have specific combinations of EDs and SUDs, it is more likely that the negative synergistic effects rather reflect that the burden of each disorder is high and may have reached a ceiling effect.

Our study has several strengths including the nationwide sample of patients with EDs, well-matched controls and long follow-up period. Healthcare is free and equally accessible in Denmark which minimises selection bias related to the ability to afford health care. In addition, since nationwide registers provide complete coverage of data on sociodemographics as well as psychiatric and somatic diagnoses that are continuously updated this limits problems with self-report or recall bias.

The present study also has some limitations. First, the registers only provide data on patients seeking hospital treatment, and we do not have information for individuals treated in primary healthcare or private institutions and individuals who avoid seeking treatment. Second, when EDs/SUDs are reported in the registers it is not common in clinical practice to use sub-coding and therefore knowledge about the severity of the disorders or recovery from them is only available for a fraction of the patients. Third, the diagnostic nomenclature does not inform us about the mode of use (inhalation, oral or intravenous administration of the drug) and specific type of drug within each hard drugs category and whether the drug was prescribed or accessed from the black market. Fourth, we had to group all hard drugs into one category. The reason for this is that in the registers providing data on SUDs, many study participants were coded as using more than one type of hard drug at the same time or as using multiple substances and other psychoactive substances (F19), depending on the clinical tradition of applying the diagnostic nomenclature. Moreover, numerous study participants with SUDs were identified through the Register of Substance Abusers in Treatment which covers the treatment of illegal drugs, and in this register, many of them were coded as treated for addiction to several illegal drugs.

Another limitation is the heterogeneity of the USED category, and it was not possible to distinguish between subtypes because the ICD-10 criteria do not permit this. Also, we only examined broad categories of somatic diseases, which restricts conclusions about severity and burden of diseases. Furthermore, we were unable to adjust the analyses for nicotine use disorder (NUD; ICD-10 code F17) and in Denmark a rather large proportion of the population are smokers. In contrast to all other SUDs, the NUD diagnosis has not, until recently, been systematically assessed in clinical practice since the Danish health care system did not offer treatment for this disorder as standard procedure. Therefore, NUD is seldomly assessed in register-based studies as it is considered a poor indicator of how many people have developed the disorder (Schmidt *et al*., 2019). Lastly, since our ED cohort was relatively young, no conclusions can be drawn regarding how SUDs may impact the risk of somatic diseases among older ED patients.

In conclusion, this study presents novel evidence that SUDs have non-synergistic but not synergistic harmful effects on somatic disease risk among patients with different EDs, with AN and hard drugs being the most predominant factors. Since ED patients often have concurrent SUD and both types of psychiatric categories are adding to somatic disease risk, it is important to monitor and treat ED patients for SUD comorbidity to prevent exacerbated physical damage in this vulnerable population.

## Data Availability

The data that support the findings of this study are available from the corresponding author [AIM], upon reasonable request.

## Footnotes

1 The ICD-9 has never been implemented in Denmark.

## Supplementary material

For supplementary material accompanying this paper visit https://doi.org/10.1017/S204579602200052X. click here to view supplementary material
