# Supplementary material for: Risk of somatic diseases in patients with eating disorders: the role of comorbid substance use disorders
Source: Epidemiol Psychiatr Sci. 2022 Oct 17;31:e73. doi: 10.1017/S204579602200052X (PMC9583632; doi:10.1017/S204579602200052X)
Supplement: Supplementary file 1 [file S204579602200052Xsup001.docx]

**SUPPLEMENTARY MATERIALS**

**Contents**

File S1….……………………………………………………………………………………….Page 1

Table S1………………………………………………………………………...........................Page 3

Table S2………………………………………………………………………………………...Page 4

Table S3………………………………………………………………………………………...Page 6

Figure S1………………………………………………………………………………………..Page 7

**FILE S1**

**Recategorized variables**

***Highest achieved education***

Data on highest achieved education at the index date was obtained from the Danish Education Registers.^1^ The original categories for the variable can be found on the following link:

<https://www.dst.dk/da/Statistik/dokumentation/Times/moduldata-for-uddannelse-og-kultur/hfaudd>.

We reclassified the variable into four groups corresponding to the following levels of the International Standard Classification of Education (ISCED 2011): 0–2 (pre-primary, primary, and lower secondary education), 3–4 (upper secondary education), 5–6 (short-cycle tertiary/bachelor’s degree or equivalent) and 7–8 (master’s degree or equivalent /doctorate or equivalent). The reclassification was defined and provided by the central authority on Danish Health Statistics (Statistics Denmark).

***Employment status***

Data on employment status at the index date was obtained from the Employment Classification Module of the Danish Income Statistics Register.^2^ In the register, individuals are classified by occupation based on their most important source of income of the year. It is determined whether a person is employed, unemployed or outside the labour force on a yearly basis. The original categories can be found on the following link: <https://www.dst.dk/da/Statistik/dokumentation/Times/personindkomst/socio13>.

We recategorized the variable into Employed (full-time or part-time), Unemployed Student (≥ 18 years), Retired, and Unknown.

SOCIO13<200 =employed

Socio13 in 210, 220, 330 unemployed

Socio13 in 420, 310 then student

Socio13 in 321, 322, 323 then retired

Other>18 years other/missing (own category)

Children at match time were not considered in this classification.

***Cohabiting status***

Information on cohabiting status at the index date was accessed from the Danish Civil Registration System.^3^ The original categories for the variable can be found on the following link: <https://www.dst.dk/da/Statistik/dokumentation/Times/cpr-oplysninger/familier-og-husstande/familie-type>)

We classified 1, 2, 3 ,4, 7, 8 to cohabiting, 5, 10, 9 to single, <18 years to child.

1. Jensen VM, Rasmussen AW. Danish Education Registers. Scand J Public Health 2011;39(7 Suppl):91-4. doi:10.1177/1403494810394715

2. Baadsgaard M, Quitzau J. Danish registers on personal income and transfer payments. Scand J Public Health 2011;39(7_suppl):103-105. doi:10.1177/1403494811405098

3. Pedersen CB. The Danish Civil Registration System. Scand J Public Health 2011;39(7 Suppl):22-5. doi:10.1177/1403494810387965

Table S1. Diagnostic crossover in patients with eating disorders

| *ED diagnosis* | Frequency (percent) |
| --- | --- |
| AN patients (n = 8,108) | |
| AN only | 5,719 (70.5%) |
| AN, then BN | 434 (5.4%) |
| AN, then USED | 1,624 (20%) |
| AN, then BN and USED | 331 (4.1%) |
| BN patients (n = 5,485) | |
| BN only | 4,265 (77.8%) |
| BN, then AN | 285 (5.2%) |
| BN, then USED | 702 (12.8%) |
| BN, then AN and USED | 233 (4.2%) |
| USED patients (7,166) | |
| USED only | 5,250 (73.3%) |
| USED, then AN | 1,159 (16.2%) |
| USED, then BN | 566 (7.9%) |
| USED, then AN and BN | 191 (2.6%) |
| Abbreviations. AN = anorexia nervosa; BN = Bulimia nervosa; ED = eating disorder; USED = unspecified eating disorder | |

Table S2. Comparison of sociodemographic and clinical characteristics across eating disorder types

|  | AN patients  (N=8,108) | | BN patients  (N=5,485) | | USED patients  (N=7,166) | |  |
| --- | --- | --- | --- | --- | --- | --- | --- |
| **Sociodemographic characteristics** | | | | | | | |
|  | N | % | N | % | N | % | p |
| Age group^a^ |  | |  | |  | | <0.001 |
| <18 years | 4,117 | 50.8 | 836 | 15.2 | 3,107 | 43.4 |  |
| 18-30 years | 3,605 | 44.5 | 4,085 | 74.0 | 3,295 | 46.0 |  |
| >30 years | 386 | 4.8 | 591 | 10.8 | 764 | 10.7 |  |
| Sex^a^ |  | |  | |  | | <0.001 |
| Male | 514 | 6.3 | 127 | 2.3 | 774 | 10.8 |  |
| Female | 7,594 | 93.7 | 5,358 | 97.7 | 6,392 | 89.2 |  |
| Ethnicity^a^ |  | |  | |  | | <0.001 |
| Danish | 7,694 | 94.9 | 5,188 | 94.6 | 6,681 | 93.2 |  |
| Immigrant or descendent | 414 | 5.1 | 297 | 5.4 | 485 | 6.8 |  |
| Cohabiting status |  | |  | |  | | <0.001 |
| Cohabiting | 1,847 | 22.8 | 1,825 | 33.3 | 1,784 | 24.9 |  |
| Living alone | 2,139 | 26.4 | 2,820 | 51.5 | 2,270 | 31.7 |  |
| Under age 18 and living with a caregiver | 4,117 | 50.8 | 836 | 15.3 | 3,107 | 43.4 |  |
| Highest achieved education |  | |  | |  | | <0.001 |
| Primary/unknown | 2,351 | 29.2 | 287 | 5.3 | 1,821 | 25.5 |  |
| Lower secondary | 3,968 | 49.2 | 2,420 | 44.4 | 3,482 | 48.7 |  |
| Upper secondary or higher | 1,745 | 21.6 | 2744 | 50.3 | 1,841 | 25.8 |  |
| Employment status at the index date |  |  |  |  |  |  | <0.001 |
| Employed | 1,179 | 14.5 | 1.684 | 30.7 | 1,258 | 17.6 |  |
| Unemployed | 675 | 8.3 | 701 | 12.8 | 921 | 12.9 |  |
| Other | 6,254 | 77.1 | 3,100 | 56.5 | 4.987 | 69.6 |  |
|  | Mean | SD | Mean | SD | Mean | SD | p |
| Age (years)^a^ | 18.8 | 5.7 | 23.0 | 5.7 | 20.3 | 7.2 | <0.001 |
| **Substance use disorder characteristics** | | | | | | | |
|  | N | % | N | % | N | % | p |
| Any SUD | 676 | 8.3 | 602 | 11.0 | 815 | 11.4 | <0.001 |
| Alcohol abuse/dependence | 305 | 3.8 | 332 | 6.1 | 345 | 4.8 | <0.001 |
| Cannabis abuse/dependence | 298 | 3.7 | 194 | 3.5 | 394 | 5.5 | <0.001 |
| Hard drug abuse/dependence^b^ | 299 | 3.7 | 281 | 5.1 | 387 | 5.4 | <0.001 |
| Timing of SUD diagnosis |  | |  | |  | | 0.004 |
| SUD predates ED by more than 1 year | 131 | 19.4 | 109 | 18.1 | 195 | 23.9 |  |
| SUD and ED diagnosed within the same year | 144 | 21.3 | 154 | 25.6 | 226 | 27.7 |  |
| ED predates SUD by more than 1 year | 401 | 59.3 | 339 | 56.3 | 394 | 48.3 |  |
|  | Mean | SD | Mean | SD | Mean | SD | p |
| Age at first SUD diagnosis (years) |  | |  | |  |  |  |
| Alcohol abuse/dependence | 26.6 | 8.1 | 29.1 | 7.6 | 26.6 | 7.7 | <0.001 |
| Cannabis abuse/dependence | 22.4 | 5.5 | 23.7 | 5.8 | 22.2 | 5.8 | 0.008 |
| Hard drug abuse/dependence | 23.0 | 6.3 | 25.2 | 7.0 | 23.6 | 6.3 | <0.001 |
| **ICD-10 somatic disorders** | | | | | | | |
|  | N | % | N | % | N | % | p |
| At least one somatic disorder after the index date | 7,408 | 91.4 | 5180 | 94.4 | 6,564 | 91.6 | <0.001 |
| Infectious diseases (A00-B99) | 950 | 11.7 | 710 | 12.9 | 810 | 11.3 | 0.015 |
| Neoplasms (C00-D48) | 675 | 8.3 | 594 | 10.8 | 518 | 7.2 | <0.001 |
| Haematological diseases (D50-D89) | 252 | 3.1 | 164 | 3.0 | 217 | 3.0 | 0.92 |
| Endocrine diseases (E00-E90) | 1,038 | 12.8 | 759 | 13.8 | 1060 | 14.8 | 0.002 |
| Neurological diseases (G00-H95) | 1,401 | 17.3 | 1,128 | 20.6 | 1,395 | 19.5 | <0.001 |
| Circulatory diseases (I00-I99) | 629 | 7.8 | 539 | 9.8 | 516 | 7.2 | <0.001 |
| Respiratory diseases (J00-J99) | 1,229 | 15.2 | 866 | 15.8 | 1,135 | 15.8 | 0.44 |
| Gastrointestinal diseases (K00-K93) | 1,940 | 23.9 | 1,454 | 26.5 | 1,795 | 25.0 | 0.003 |
| Dermatological diseases (L00-L99) | 1,044 | 12.9 | 739 | 13.5 | 837 | 11.7 | 0.007 |
| Musculoskeletal diseases (M00-M99) | 2,402 | 29.6 | 1,732 | 31.6 | 2,038 | 28.4 | <0.001 |
| Genitourinary diseases (N00-N99) | 2,440 | 30.1 | 2063 | 37.6 | 2,087 | 29.1 | <0.001 |
| **Mortality** | | | | | | | |
|  | N | % | N | % | N | % | p |
| Deaths during follow-up | 115 | 1.4 | 53 | 1.0 | 126 | 1.8 | <0.001 |
|  | Mean | SD | Mean | SD | Mean | SD |  |
| Age at death | 30.1 | 8.5 | 30.7 | 7.7 | 30.9 | 8.7 | 0.76 |
| ^a^ Matching variable  ^b^ The hard drugs category includes heroin and other opioids, sedative-hypnotics, cocaine and other stimulants, multiple substances, and other psychoactive substances (e.g., hallucinogens, volatile solvents, and designer drugs).  ^c^ The timing of first SUD diagnosis among individuals in the control group was determined from the index date of the eating disorder patients to whom they were matched.  AN = anorexia nervosa; BN = bulimia nervosa; SD = standard Deviation; SUD = substance use disorder; USED = unspecified eating disorder | | | | | | | |

|  | | USED:  Neurological diseases | USED:  Gastrointestinal diseases | USED:  Musculoskeletal diseases | AN:  Genitourinary diseases | BN:  Genitourinary diseases |
| --- | --- | --- | --- | --- | --- | --- |
|  |  | HR (95% CI) | HR (95% CI) | HR (95% CI) | HR (95% CI) | HR (95% CI) |
| SUD | No SUD | Reference | Reference | Reference | Reference | Reference |
|  | Alcohol only | 1.68 (1.21; 2.34) | 1.72 (1.28; 2.3) | 1.72 (1.37; 2.17) | 1.49 (1.19; 1.89) | 1.74 (1.39; 2.19) |
|  | Cannabis (w/wo alcohol) | 1.66 (1.23; 2.25) | 1.73 (1.33; 2.26) | 1.47 (1.15; 1.87) | 1.61 (1.29; 2.01) | 1.32 (0.94;1.84) |
|  | Hard drugs (w/wo alcohol or cannabis) | 1.71 (1.36; 2.15) | 1.84 (1.52; 2.24) | 1.77 (1.48;2.11) | 2.02 (1.73; 2.36) | 1.54 (1.26;1.88) |
| ED | Control | Reference | Reference | Reference | Reference | Reference |
|  | ED | 1.76 (1.65;1.88) | 1.81 (1.7;1.92) | 1.49 (1.41;1.57) | 1.4 (1.33; 1.47) | 1.36 (1.29;1.43) |
| Interaction | No SUD*ED | Reference | Reference | Reference | Reference | Reference |
|  | Alcohol only*ED | 0.78 (0.51; 1.2) | 0.87 (0.6; 1.26) | 0.76 (0.54; 1.05) | 0.63 (0.45; 0.89) | 0.53 (0.39; 0.73) |
|  | Cannabis (w/wo alcohol)*ED | 0.73 (0.48; 1.1) | 0.57 (0.38; 0.83) | 0.71 (0.5;1) | 0.81 (0.57; 1.15) | 1.08 (0.69; 1.69) |
|  | Hard drugs (w/wo alcohol or cannabis)*ED | 0.64 (0.47; 0.88) | 0.92 (0.71; 1.19) | 0.64 (0.5; 0.82) | 0.64 (0.5; 0.81) | 0.85 (0.65; 1.12) |
| AN = anorexia nervosa; BN = bulimia nervosa; ED = eating disorder; HR = hazard ration; SUD = substance use disorder; USED = unspecified eating disorder | | | | | | |

Table S3. Interactions

Figure S1. Cumulative incidents rates for being diagnosed with a somatic disorder (within 11 categories) after the index date among patients with eating disorders compared to matched controls


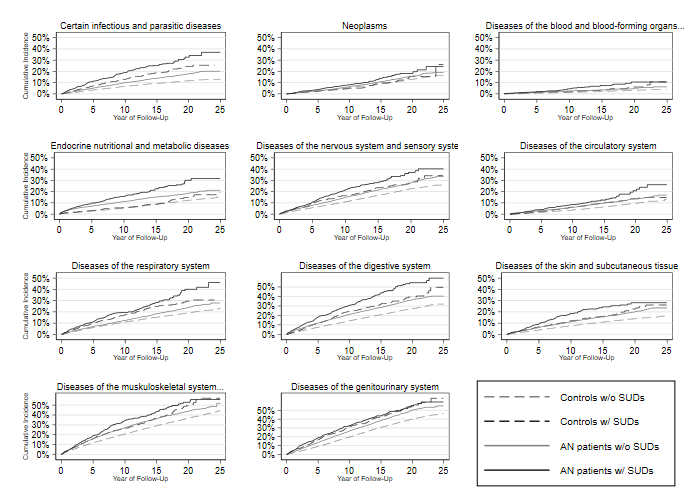


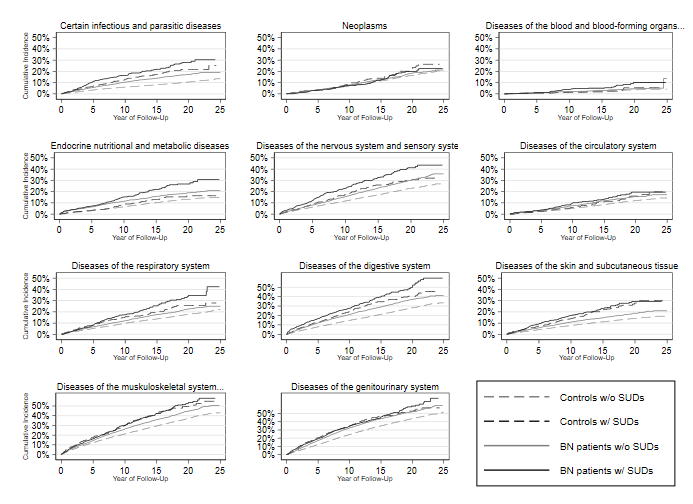

1. Cumulative incidence in 1) anorexia nervosa (AN) patients with substance use disorders (SUDs); 2) AN patients without SUDs; 3) AN control subjects with SUDs; and 4) AN control subjects without SUDs
2. Cumulative incidence in 1) bulimia nervosa (BN) patients with SUDs; 2) BN patients without SUDs; 2) BN control subjects with SUDs; and 3) BN control subjects without SUDs.
3. Cumulative incidence in 1) unspecified eating disorder (USED) patients with SUDs; 2) USED patients without SUDs; 3) USED control subjects with SUDs; and 4) USED control subjects without SUDs
